# Chondroprotective effects of *Protaetia brevitarsis seulensis* larvae as an edible insect on osteoarthritis in mice

**DOI:** 10.1002/fsn3.3706

**Published:** 2023-10-06

**Authors:** Jin Mi Chun, Hyeon‐Hwa Nam, Ji Hye Lee, Young Hye Seo, Hyo Seon Kim, Byeong Cheol Moon, Jun Hong Park

**Affiliations:** ^1^ Herbal Medicine Resources Research Center Korea Institute of Oriental Medicine Naju‐si Republic of Korea; ^2^ School of Korean Medicine Pusan National University Busan‐si Republic of Korea

**Keywords:** chondroprotective effects, edible insect, osteoarthritis, *Protaetia brevitarsis seulensis* larva

## Abstract

Osteoarthritis (OA) is a common chronic joint inflammatory disease characterized by progressive destruction of the articular cartilage, bone remodeling, and excessive chronic pain. Most therapeutic approaches do not rescue the progression of OA effectively or provide relief of symptoms. *Protaetia brevitarsis seulensis* larva (PBSL), which is attracting attention, is an edible insect with very high nutritional value and herbal medicine for the treatment of blood stasis, hepatic disease, and various inflammatory diseases. However, the effect of PBSL on OA has not yet been investigated. This study aimed to demonstrate the effects of PBSL water extract on the progression of OA using monosodium iodoacetate (MIA)‐induced mice and SW1353 chondrocytes or murine macrophages. We injected MIA into the intraarticular area of mice following pretreatment with either saline or PBSL (200 mg/kg) for 2 weeks, and then locomotor activity, microcomputed tomography and histopathological analysis, quantitative reverse transcriptase–polymerase chain reaction analysis, and western blot analysis were performed. To determine the molecular effects of PBSL, we used interleukin‐1β (IL‐1β)‐induced SW1353 chondrosarcoma or lipopolysaccharide (LPS)‐stimulated macrophages. Pretreatment with PBSL diminished the symptoms of OA. Physical activity, articular cartilage damage, and the generation of microfractures were rescued by pretreatment with PBSL in the mouse model. Pretreatment with PBSL suppressed the progress of OA through the regulation of articular cartilage degradation genes and inflammation in both in vivo and in vitro models. Our results demonstrated that PBSL has value as edible insect that can be used in the development of functional foods for OA.

## Graphical abstract

Pretreatment with *Protaetia brevitarsis seulensis* larvae (PBSL) diminished the symptoms of OA. Physical activity, articular cartilage damage, and the generation of microfractures were rescued by pretreatment with PBSL in the mouse model. Pretreatment with PBSL suppressed the progress of OA through the regulation of articular cartilage degradation genes and inflammation in both in vivo (monosodium iodoacetate‐induced mice) and in vitro (SW1353 and RAW264.7) models.

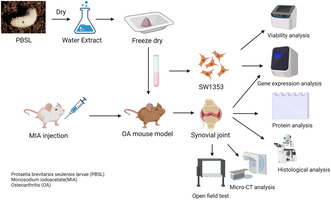

## 1 INTRODUCTION

Osteoarthritis (OA) is a degenerative joint disease characterized by chronic joint inflammation and articular cartilage degradation (Sandell & Aigner, 2001). The prevalence of OA has steadily increased worldwide, along with the increase in the elderly population (Shane Anderson & Loeser, 2010; Veronese et al., 2020). Although numerous studies have suggested that many factors are associated with the onset of OA, the underlying causes of OA are complex. Recent evidence suggests that OA is characterized by multiple risk factors, including aging, obesity, loss of normal joint function, and low‐grade systemic inflammation (Katz et al., 2021; Sokolove & Lepus, 2013). OA remains difficult to treat, and research on its definition, risk factors, causes, and pathophysiology is ongoing.

The goal of the present OA study was to search for new therapeutic strategies that could prevent, reduce, or halt disease progression, or reverse existing damage to joints. Although current treatments for OA remain limited, medical management includes nonpharmacological, pharmacological, and multiple combination therapies (Martel‐Pelletier et al., 2016). Conservative treatment options include therapeutic exercise, manual therapy, patient education, and pharmacological agents, and the effectiveness of various methods have been reported (Sánchez Romero, et al., 2020; Sánchez Romero, González‐Zamorano, et al., 2021; Sinatti et al., 2022). In addition, studies on pharmacological treatment efficacy as well as other treatments and new pain relief products are being actively reported.

The prevention or treatment of OA is of utmost importance, as OA patients experience disability and diminished quality of life due to knee inflammation and severe pain. One of the pathogeneses of OA is an inflammatory response, which contributes to the onset and progression of OA (Khan et al., 2017; Lin et al., 2018). During the pathological progression of OA, the production of various inflammatory cytokines such as IL‐1 or TNFα and the breakdown of extracellular matrices in joint tissues and body fluids interact with chondrocytes and induce osteoarthritic cartilage damage (Aigner & McKenna, 2002). These inflammatory cytokines and proteases are involved in the destruction of cartilage matrix components while sustaining inflammation (Goldring & Otero, 2011). Nitric oxide (NO) in macrophages is mostly produced by inducible NO synthase (iNOS), which is a major factor in the inflammatory response (Tang et al., 2020). In OA, NO is produced in response to synovial inflammation and affects the balance of cartilage matrix degradation and repair (Lee et al., 2019), and is involved in the inhibition of collagen and proteoglycan synthesis and induction of chondrocyte apoptosis (Ostojic et al., 2017). Therefore, a pharmacological approach that reduces the degradation of OA articular cartilage and synovial inflammation is needed for the management of OA.

Current pharmacological treatments for OA are primarily focused on relieving symptoms. For example, treatments include the injection of analgesics in the intraarticular area or nonsteroidal anti‐inflammatory drug treatment (Hermann et al., 2018). However, chronic use of these agents can cause a wide range of side effects, including toxicity and risk (Cooper et al., 2019). Studies on the development of new pain‐relief products using natural products are ongoing.

Recent scientific evidence indicates that the nutritional quality of edible insects is equivalent to, and sometimes more than, the nutritional quality of animal foods (Orkusz, 2021). One of the edible insects, *Protaetia brevitarsis seulensis* larva (PBSL), is currently listed as a food resource by the Ministry of Food and Drug Safety. In traditional East Asian medicine, it has been used for the treatment of blood stasis, impediment disease, hepatic disease, and various inflammatory diseases. It is also used to reduce swelling, fever, and pain, as well as to treat other diseases (Deyrup et al., 2021). Recent reports have shown that PBSL has various pharmacological properties, including antioxidant, anti‐inflammatory, and antithrombotic effects (Lee et al., 2017; Yoon et al., 2020). Previously, we have demonstrated that the water extract of PBSL showed neuroprotective effects on trimethyltin‐induced seizures and hippocampal neurodegeneration both in vitro and in vivo, and chemical profiling of this extract identified six major compounds (e.g., adenine, adenosine, benzoic acid, hypoxanthine, inosine, and uridine). In addition, recent studies have shown that treatment with PBSL extract or its derived products improved osteogenic differentiation in human bone marrow‐derived mesenchymal stem cells and exerted hepatoprotective effects (Ganguly et al., 2020; Im et al., 2018). However, the functionality of PBSL in chronic joint inflammation and OA remain unclear. In order to confirm the health benefits of OA of PBSL, which has recently been attracting attention, we extended our previous observations to demonstrate the effects of PBSL on OA. In this study, we used a monosodium iodoacetate (MIA)‐induced OA mouse model and interleukin‐1β (IL‐1β)‐induced SW1353 chondrosarcoma cells or lipopolysaccharide (LPS)‐stimulated macrophages in vitro models to study the effects of PBSL on OA.

## 2 MATERIALS AND METHODS

### 2.1 Preparation of aqueous extract of PBSL

Authentication of *P. brevitarsis* and PBSL was performed as previously described (Lee et al., 2021). Briefly, dried PBSL was purchased from a commercial medicinal company (Kwang Myung Dang Co). The authentication of *P. brevitarsis* was confirmed by morphological identification and genetic analysis using DNA barcoding analysis, and the specimen was deposited at the Korean Herbarium of Standard Herbal Resources (No. 2‐18‐0111). To prepare the crude extract, the dried sample (887.4 g) was refluxed in distilled water for 3 h, and the extract was concentrated under reduced pressure (yield: 27.4%). Before use *in* in vitro and in vivo experiments, the lyophilized powder was dissolved in phosphate‐buffered saline or 0.25% carboxymethyl cellulose.

### 2.2 Experimental design

C57BL/6J female mice (7 weeks of age) were purchased from Doo Yeol Biotech, Inc. and maintained in an air‐conditioned environment with a 12 h light/dark cycle. Water and experiment diets were provided *ad libitum*. After acclimatization, mice were randomly divided into three groups at 9 weeks of age (*n* = 6–7 per group) as follows: untreated with saline (control), MIA‐injected with pretreatment of saline (MIA), and MIA‐injected with pretreatment of PBSL (MIA + PBSL). The animal study was conducted in accordance with the Committee of Laboratory Animal Care and Use, Korea Institute of Oriental Medicine (KIOM 20‐028).

**FIGURE 1:**
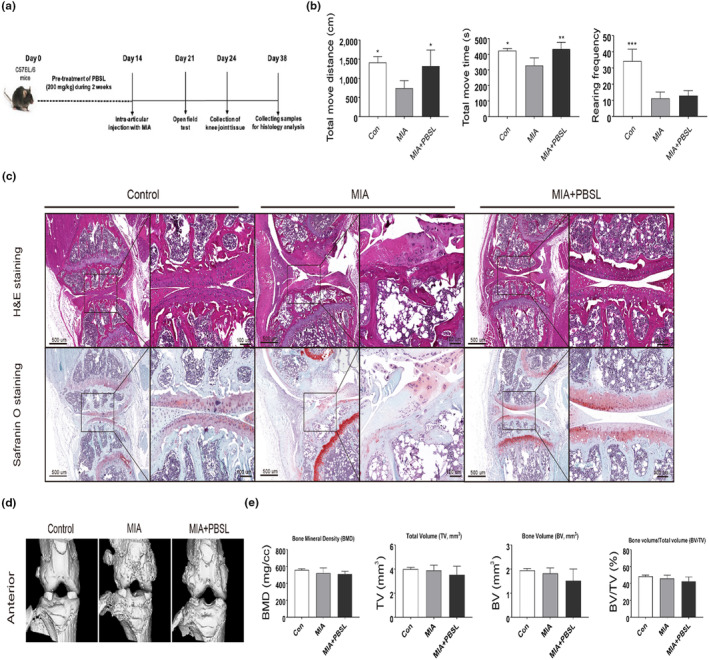
Effects of *Protaetia brevitarsis seulensis* larva (PBSL) on osteoarthritis (OA) symptoms in monosodium iodoacetate (MIA)‐induced OA mouse models. (a) The experimental scheme used to induce OA and administer oral pretreatment of PBSL. (b) At 7 days post‐MIA injection, locomotor activity was monitored using an open‐field test with an automated monitoring system. Data were analyzed with one‐way analysis of variance (ANOVA) (*n* > 3 per group, *p < .05, **p < .01 ***p < .001 vs. MIA group). (c) Histological analysis of mouse knee joint sections was stained with hematoxylin and eosin and Safranin O‐fast green (insets show higher magnification, 100×). Scale bars are indicated. (d) Microcomputed tomography (CT) images of mouse knee joint tissue were scanned using an in vivo micro‐CT imaging system. (e) Quantification of micro‐CT analysis image was analyzed using AccuCT micro‐CT analysis software. Data were analyzed using one‐way ANOVA (*n* = 3 per group). Con, untreated with saline; MIA, MIA‐injected with pretreatment of saline; MIA + PBSL, MIA‐injected with pretreatment of PBSL mice.

The experimental protocol is shown in Figure 1a. First, to investigate the preventive effect of PBSL on OA, mice were orally administered PBSL (200 mg/kg) for 2 weeks from day 1. On day 14, 0.9% saline (0.75 μg/10 μL) dissolved in MIA (I2512, Sigma) dissolved at 0.75 μg/10 μL in 0.9% saline was injected into the intraarticular area to develop the MIA‐induced OA mouse model (Pitcher et al., 2016). Seven days post‐MIA injection (day 21), the locomotor activity was assessed using an automated monitoring system (TruScan Activity Monitor version 2.04, Coulbourn Instruments). Mice were placed in a square arena (40 × 40 × 40 cm) with transparent walls and allowed to navigate freely for 10 min in a soundproof room. The system was designed to enable the separate monitoring of horizontal (XY‐move time) and vertical activity (rearing). The parameters measured were total distance moved (cm), total movement time (s), and rearing frequency. On day 24, samples of knee joint tissue were collected from the mice for a mechanism study. Finally, the remaining samples were collected for histological analysis on day 38.

### 2.3 Microcomputed tomography (CT) and histopathological analysis

At the end of the experiment, mice were euthanized. The right knee tissues were dissected, skin and extra tissue were removed, and were fixed in 10% neutral buffered formalin for micro‐CT scanning and/or evaluation. The knee tissues were scanned using Quantum FX micro‐CT imaging system (PerkinElmer) to evaluate structural alterations in the subchondral bone architecture. The acquired image data were reconstructed using the 3D Viewer software available within the imaging system and analyzed using the Analyze 12.0 Viewer software (PerkinElmer).

Following micro‐CT scanning, the tissues were sequentially decalcified, dehydrated, embedded in paraffin, and serially sectioned for histological analysis. Decalcification was performed following the manufacturer's instructions (D0818, Sigma). The paraffin blocks were sectioned at a thickness of 5 μm and sections were deparaffinized in xylene and hydrated by using graded ethanol (Kim et al., 2019). The prepared sections were stained with hematoxylin and eosin (H&E) and Safranin O‐fast green (Safranin O) to observe changes in joint cells, matrices, and proteoglycans in the articular cartilage (Kim et al., 2019). Histological changes were analyzed using a PANNORAMIC desk instrument (3DHISTECH).

### 2.4 Cell culture

Human chondrosarcoma SW1353 and murine macrophage RAW264.7 cells were purchased from the American Type Culture Collection (Manassas, VA, USA) and maintained in Dulbecco's modified Eagle's medium (DMEM) supplemented with 1% penicillin/streptomycin and 10% fetal bovine serum (FBS) at 37°C in a 5% CO_2_ incubator (Park et al., 2015).

### 2.5 MTS assay

For the cell viability assay, SW1353 cells were seeded in a 96‐well plate (1 × 10^4^ cells/well). After 24 h, the cells were pretreated with PBSL (100, 200, and 400 μg/mL) for an additional 2 h. Cells were then stimulated with IL‐1β (20 ng/mL) (Pepro Tech) for 24 h. Cell viability was evaluated using the Celltiter 96® aqueous one solution cell proliferation assay (MTS) according to the manufacturer's instructions (Promega). After 1 h, the MTS assay was performed to assess cell viability, and the absorbance at 490 nm was measured using a microplate reader (Spectra‐Max MiniMax 300, Molecular Devices).

### 2.6 CCK‐8 assay

RAW264.7 cells were cultured in 96‐well plates (2 × 10^4^ cells/well) and treated with different concentrations of PBSL (100, 200, and 400 μg/mL) and 100 μM of compounds (benzoic acid, adenosine, uridine, hypoxanthine, adenine, and inosine), followed by cotreatment with LPS (100 ng/mL). Cell viability was determined using a cytotoxicity assay kit (Cell Counting Kit‐8, Dojindo Molecular Technologies), and the absorbance was measured at 450 nm using a microplate reader (SpectraMax MiniMax 300, Molecular Devices).

### 2.7 Measurement of NO synthesis

To identify the anti‐inflammatory effects of PBSL or PBSL‐derived compounds, NO levels in the LPS‐induced RAW264.7 cells were measured by Griess assay as described methods (Yamanishi et al., 2009). The cells were seeded in 96‐well plates (2 × 10^4^ cells/well) and treated with different concentrations of PBSL (100, 200, or 400 μg/mL) and 100 μM of compounds (benzoic acid, adenosine, uridine, hypoxanthine, adenine, and inosine) followed by cotreatment with LPS (100 ng/mL). The supernatant (50 μL) was mixed with 50 μL of 1% sulfanilamide and 0.1% N‐1‐naphthylethylenediamine and incubated at 25°C for 10 min. The absorbance was measured at 540 nm using a microplate reader (SpectraMax MiniMax 300). NO production in the samples was calculated using a standard curve.

### 2.8 Quantitative reverse transcriptase–polymerase chain reaction (qRT‐PCR) analysis

Quantitative reverse transcriptase–polymerase chain reaction (qRT‐PCR) analysis was performed as previously described (Xue et al., 2017). Total RNA was isolated from the mouse knee tissues or treated cells using Easy‐Spin Total RNA Extraction Kit (iNtRON Biotechnology). Complementary DNA was prepared using SuperScript4 cDNA Synthesis Kit (Thermo Fisher Scientific). qPCR analysis was performed using CFX96 real‐time PCR detection systems (Bio‐Rad) using SYBR Green Master MIX (Thermo Fisher Scientific). The primer sequences used are listed in Table S1.

### 2.9 Western blotting (WB) analysis

Western blotting (WB) analysis was performed as previously described (Lee et al., 2022). Proteins were prepared from the knee tissue or treated cells using the M‐PER Mammalian Protein Extraction Reagent (Thermo Fisher Scientific) on ice for 30 min. Proteins (30 μg) in the prepared samples were then separated by 10% SDS‐PAGE and transferred to nitrocellulose membranes for WB analysis. The membranes were blocked using 5% skim milk and incubated overnight at 4°C with primary antibodies against β‐actin (Sigma), followed by the corresponding horseradish peroxidase‐conjugated secondary antibodies (Santa Cruz Biotechnology) for 1 h at room temperature. Each protein was detected with a corresponding antibody listed in Table S2. The membranes were then treated with an ECL detection reagent (Thermo Fisher Scientific). WB images were detected using the ChemiDoc imaging system (Bio‐Rad). Quantification of the bands was performed using Image Lab 6.1 software (Bio‐Rad).

### 2.10 Statistical analysis

Statistical analysis was performed using the GraphPad Prism 7 program (GraphPad Software). The results are presented as the mean ± standard deviation (SD). To determine the significance of differences between mouse or cell groups, one‐way analysis of variance, with Dunnett's multiple comparisons test was employed. The significance was set at *p < .05, **p < .01, and ***p < .001.

## 3 RESULTS

### 3.1 Pretreatment of PBSL decreased OA symptoms in the mouse model

To evaluate the effects of PBSL in MIA‐induced OA mice, locomotor activity was assessed using an open‐field automated monitoring system. Locomotor activity is one of the most well‐known behavioral parameters and is mainly used to predict the pain relief effect of compounds in models of joint inflammation and OA (Alsalem et al., 2020). The total movement distance, total movement time, and rearing frequency of MIA‐induced OA mice were significantly lower than those of the control mice (Figure 1b). Compared to the MIA‐induced OA group, PBSL‐treated mice showed a significant increase in total movement distance and total movement time, but there was no significant difference in rearing frequency. These results demonstrated that treatment with PBSL increased locomotion parameters and alleviated gait disturbance, a major symptom in the OA model.

Next, to confirm the effects of PBSL on the histological features of the mouse knee in MIA‐induced OA mice, the knee joint tissues were evaluated via histological staining (H&E and Safranin O, Figure 1c). H&E staining showed MIA‐induced cartilage destruction and degeneration of the articular cartilage. MIA group showed deformity and irregularity in cartilage. Treatment with PBSL blocked deformity and irregularity of cartilage area, this result indicated that PBSL reduced the articular cartilage damage. Consistent with this, Safranin O staining results also showed remarkably reduced chondrocytes and proteoglycan. This result demonstrated that administration of PBSL for 2 weeks could reduce the destruction of cartilage compared with the MIA group; however, the PBSL‐treated group evidently exhibited rescued chondrocytes and proteoglycan. To confirm our results, we analyzed the articular structure using micro‐CT (Figure 1d). 3D reconstruction of the subchondral bone from our micro‐CT data showed that PBSL suppressed the generation of osteophytes and microfractures in the subchondral bone. Microfracture generation in the subchondral bone is the main bone‐related source of pain in OA (Salaffi et al., 2014). These results were consistent with the results of the locomotor activity. Furthermore, our micro‐CT analysis distinctly showed that PBSL did not affect bone mineral density, total volume, or bone volume (Figure 1e). Taken together, these results indicate that PBSL has a protective effect against MIA‐induced articular cartilage damage and bone remodeling.

### 3.2 Pretreatment with PBSL affected the expression of matrix metalloproteinases (MMPs) and their inhibitors on the progress of OA in the knee joint tissue

MMPs and their inhibitors, which control degradation of the articular surface and induce remodeling of the extracellular matrix, play an important role in the progression of OA and regulate the release of cell signaling receptor molecules (Jayakumar et al., 2020; Raeeszadeh‐Sarmazdeh et al., 2020). To determine whether treatment with PBSL affects the level of MMP family members and their inhibitors, we examined their levels in mouse knee tissue using qPCR or WB analysis.

**FIGURE 2:**
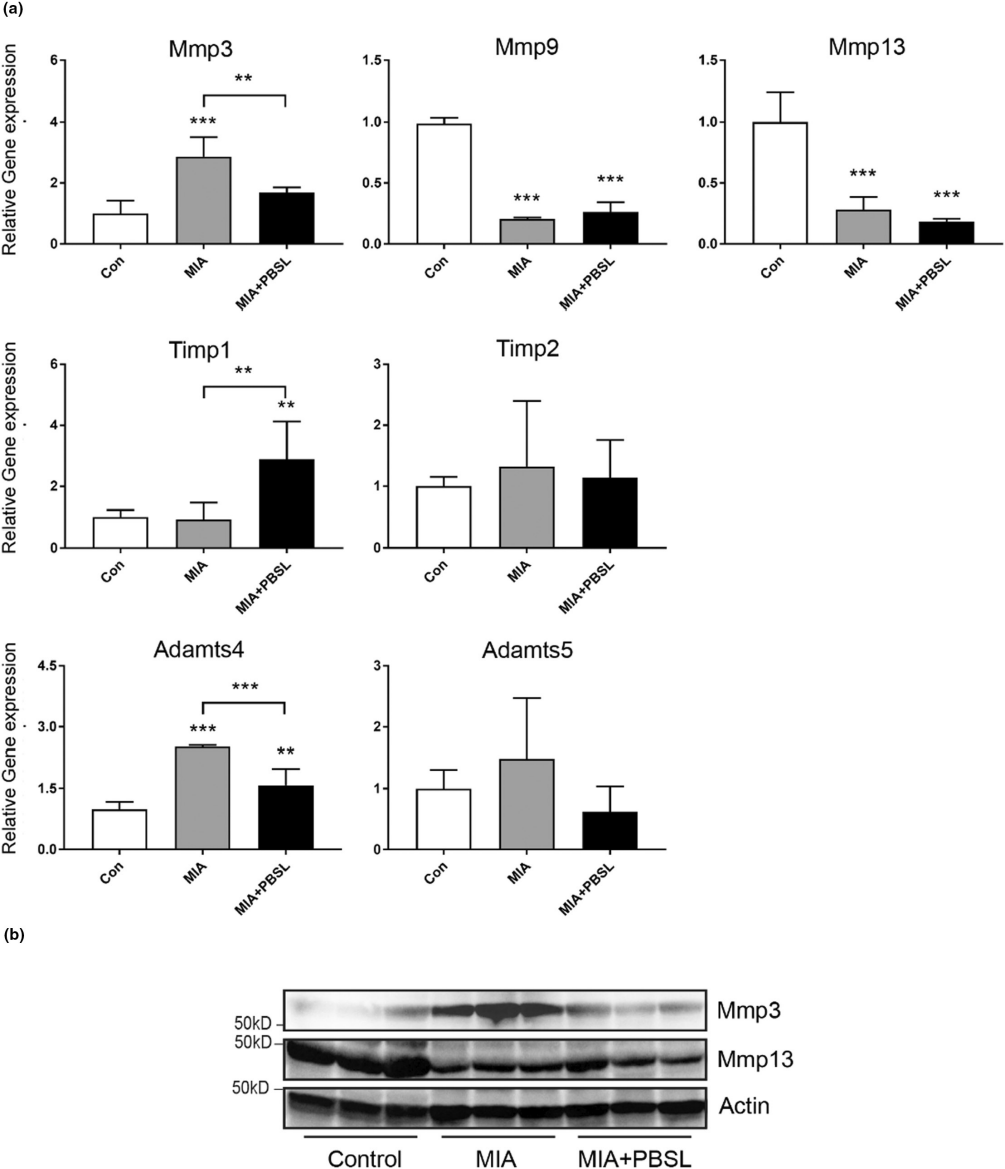
Effects of *Protaetia brevitarsis seulensis* larva (PBSL) on levels of MMPs and their inhibitors in monosodium iodoacetate (MIA)‐induced osteoarthritis mice knee tissue. (a) qPCR analysis was performed for gene expression of MMPs and their inhibitors in mouse knee joint tissue. Data were analyzed using one‐way analysis of variance (ANOVA) (*n* > 3 per group, *p < .05, ***p < .001 vs. Control group, ***p < .001 vs. MIA alone group). (b) Western blot analysis of the knee joint tissue extracts was performed for the protein expression of matrix metalloproteinase (MMP)3 and MMP13. Con, untreated with saline; MIA, MIA‐injected with pretreatment of saline; MIA + PBSL, MIA‐injected with pretreatment of PBSL mice.

We found that MIA induced the expression of MMP3 and A disintegrin and metallo‐peptidase with thrombospondin type 1 motif (ADAMTs) 4 compared with the control group. The expression of MMP3 and ADAMTs4 was significantly suppressed in the PBSL‐treated group compared with that in the MIA‐induced OA mice (Figure 2a). More interestingly, the treatment of PBSL significantly induced the expression of TIMP metallo‐peptidase inhibitor 1 (TIMP1). WB analysis revealed that the protein level of MMP3 was decreased in the PBSL‐treated group compared with that in the MIA‐induced OA group. Supported by the qRT‐PCR results, the protein level of MMP13 was decreased in the MIA‐induced OA and PBSL‐treated group compared with that of the control group (Figure 2b). These results suggest that PBSL is involved in the regulation of MMPs and their inhibitors in the progression of OA in the mouse knee joint tissues.

### 3.3 Pretreatment of PBSL affected the expression of MMPs and their inhibitors in the in vitro chondrocyte OA model

**FIGURE 3:**
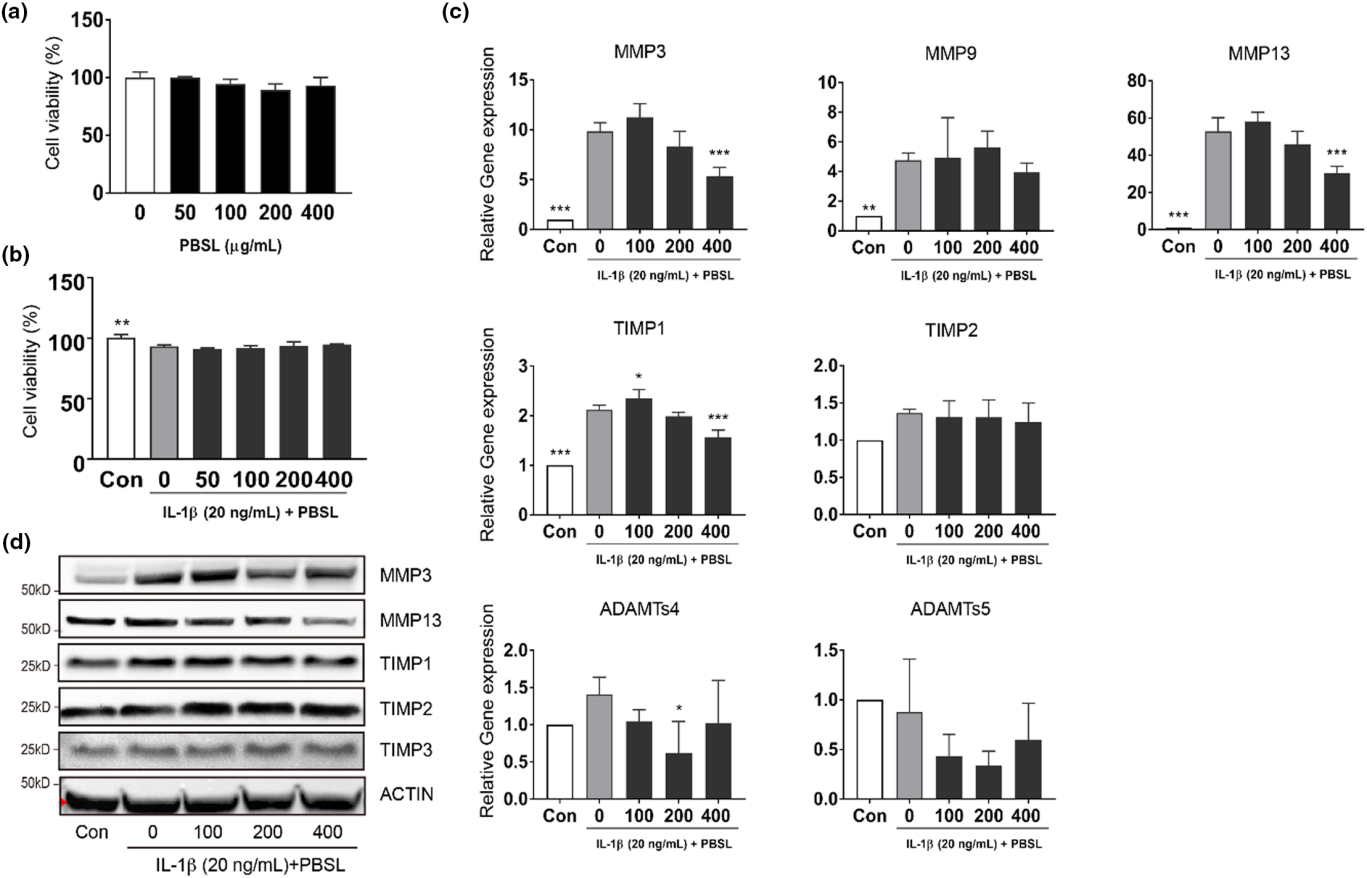
Effects of *Protaetia brevitarsis seulensis* larva (PBSL) on the expression of matrix metalloproteinases (MMPs) and their inhibitors in interleukin (IL)1β‐induced SW1353 cells. (a) Cell viability was determined via MTS assay. Cells were treated with various concentrations of PBSL (50–400 μg/mL) for 24 h. (b) Cells were pretreated with PBSL (50–400 μg/mL) for 2 h and stimulated with IL‐1β (20 ng/mL) or IL‐1β alone for 24 h. Cell viability was determined using MTS assay. (c) Quantitative polymerase chain reaction analysis was performed for gene expression of MMPs and their inhibitors in SW1353 cells after treatment. (d) Western blot analysis was performed for protein expression of metalloproteinases and their inhibitors in SW1353 cells. Data were analyzed using one‐way analysis of variance (ANOVA) (*n* > 3 per group, *p < .05, **p < .01, ***p < .001 vs. IL‐1β alone group).

To further investigate the effects of PBSL on chondrocytes, we mimicked OA in vitro using the SW1353 cell line and IL‐1β (Vincenti & Brinckerhoff, 2001). We first confirmed the cellular viability of SW1353 cells in response to various concentrations of PBSL using the MTS assay. As expected, treatment with various concentrations of PBSL did not affect the viability of SW1353 cells (from 50 to 400 μg/mL of PBSL, Figure 3a). In addition, cotreatment with various concentrations of PBSL and IL‐1β (20 ng/mL) did not result in synergetic toxicity (Figure 3b). Next, we determined whether pretreatment with PBSL affected the expression of MMPs and their inhibitors, which are involved in the progression of OA, in SW1353 cells. The gene expression of MMPs and their inhibitors (MMP3, MMP9, MMP13, and TIMP1) significantly increased following treatment with IL‐1β. Pretreatment with PBSL significantly inhibited the mRNA expression of MMP3 and MMP13 in IL‐1β‐stimulated SW1353 cells (Figure 3c). Interestingly, their inhibition levels were dependent on the concentration of PBSL. Although TIMP1 expression was significantly increased at a low concentration of PBSL (100 μg/mL), it gradually decreased after pretreatment with PBSL in a dose‐dependent manner. ADAMTs4 expression was significantly inhibited by treatment with 200 μg/mL of PBSL; however, it did not show a constant tendency. Consistent with the gene expression results, pretreatment with PBSL suppressed the protein levels of MMP3 and MMP13 in IL‐1β‐stimulated SW1353 cells (Figure 3d). The expression of TIMP1 and TIMP2 increased at a low concentration of PBSL (100 μg/mL), which was slightly decreased by treatment with PBSL (400 μg/mL) compared to treatment with IL‐1β only. Thus, our results suggest that pretreatment with PBSL regulated the levels of OA pathological markers in IL‐1β‐stimulated SW1353 cells.

### 3.4 Pretreatment with PBSL decreased the generation of NO in RAW264.7 cells

**FIGURE 4:**
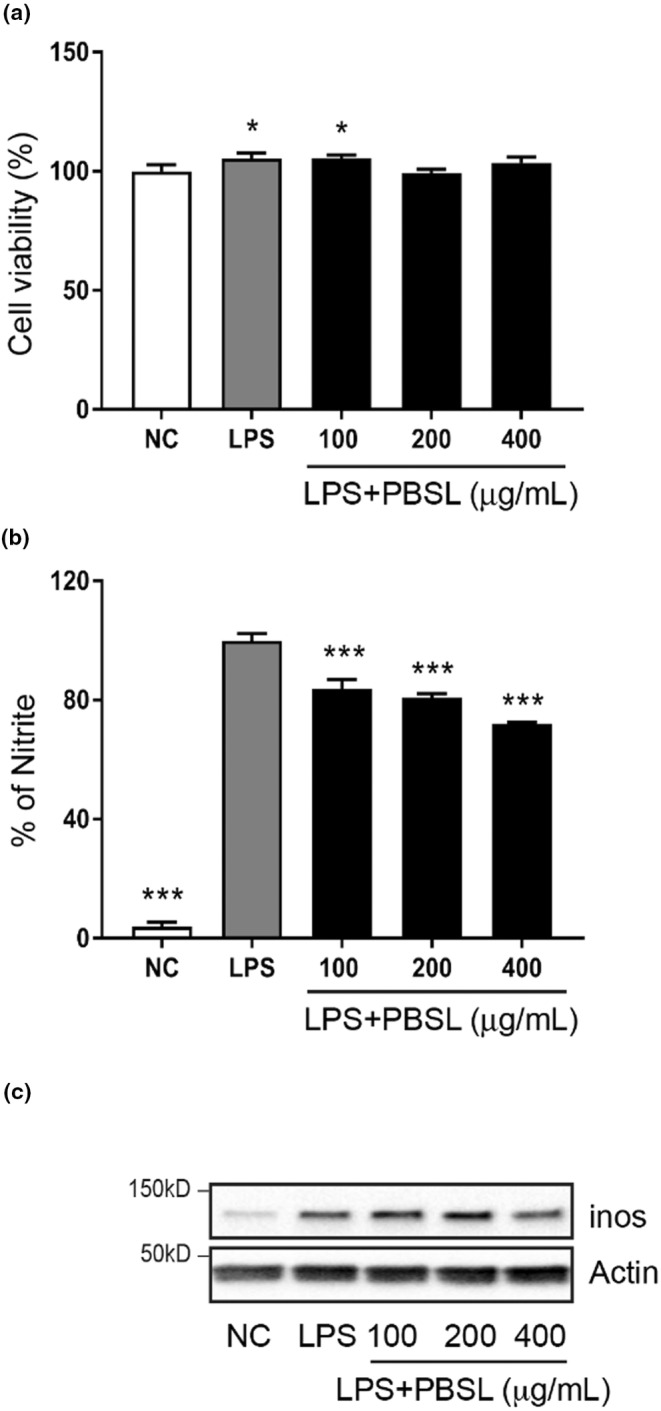
Effects of *Protaetia brevitarsis seulensis* larva (PBSL) on inflammatory mediators in RAW264.7 cells. (a) Cell viability, (b) NO synthesis, and (c) expression of the iNOS protein after treatment of PBSL in lipopolysaccharide (LPS)‐induced RAW264.7 cells. The cells were treated with PBSL (100, 200, and 400 μg/mL) and LPS (100 ng/mL) for 18 h. The analysis of NO synthesis and iNOS expression by PBSL was measured using the Griess assay and western blot analysis. Data were analyzed using one‐way analysis of variance (ANOVA) [*n* > 3 per group, *p < .05, ***p < .001 vs. NC (a) or LPS alone group (b)].

NO and iNOS are important regulators of OA, synovial inflammation, and chondrocyte apoptosis in synovial joint tissues (Leonidou et al., 2020). To confirm whether pretreatment with PBSL inhibits the generation of NO and/or iNOS, we measured the levels of NO and expression of iNOS in LPS‐induced RAW264.7 cells. As shown by the cell viability assay, there were no changes in viability after treatment with LPS and PBSL in RAW264.7 cells (Figure 4a). Importantly, the generation of NO was significantly diminished in all PBSL pretreatment groups. Furthermore, the decline in NO production was dependent on the pretreatment concentration of PBSL compared to that in the LPS‐only group (Figure 4b). WB assay showed that iNOS expression increased in the LPS‐treated group compared to that in the normal group. Although the expression of iNOS was not significantly decreased in all pretreatment groups, pretreatment with PBSL (400 μg/mL) suppressed the level of iNOS revealed by the measurement of NO levels and the WB assay, pretreatment with PBSL also suppressed the generation of NO in RAW264.7 cells.

### 3.5 Pretreatment with PBSL suppressed the expression of inflammatory cytokines and mediators in the chondrocytes or knee joint tissues

**FIGURE 5:**
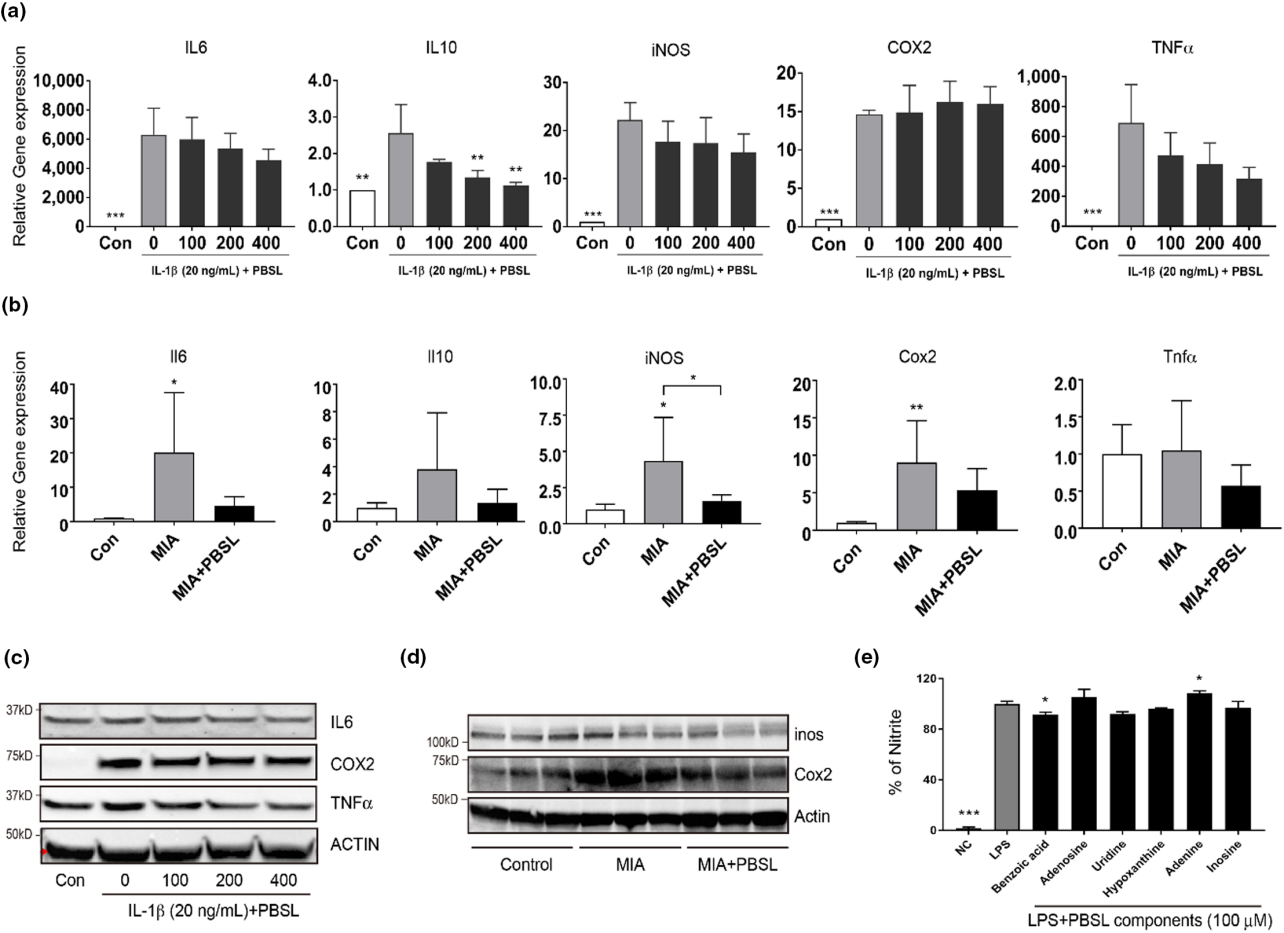
Effects of *Protaetia brevitarsis seulensis* larva (PBSL) or PBSL‐derived components on inflammatory factors in SW1353 cells or monosodium iodoacetate (MIA)‐induced mice knee tissue. (a) mRNA expression of inflammatory cytokines and mediators in SW1353 cells. Data were analyzed using one‐way analysis of variance (ANOVA) (*n* > 3 per group, **p < .01, ***p < .001 vs. IL‐1β alone group). (b) mRNA expression of inflammatory cytokines and mediators in mice knee tissue 10 days after MIA induction. Data were analyzed using one‐way ANOVA (*n* > 3 per group, *p < .05, **p < .01 vs. Control group, *p < .05 vs. MIA alone group). (c) Protein levels of interleukin‐6, cyclooxygenase 2 (COX2), and tumor necrosis factor‐α in SW1353 cells. (d) Protein levels of COX2 and iNOS in mice knee tissue 10 days post‐MIA induction. (e) Nitrite oxide (NO) synthesis by PBSL compound treatment in lipopolysaccharide (LPS)‐induced RAW264.7 cells. Cells were treated with 100 μM of PBSL compounds (benzoic acid, adenosine, uridine, hypoxanthine, adenine, and inosine) with LPS (100 ng/mL) for 18 h. Inhibition effects of components on NO synthesis were analyzed using a Griess assay. Data were analyzed using one‐way ANOVA (*n* > 3 per group, *p < .05 vs. LPS alone group).

Inflammatory mediators play an important role in the pathological progression of OA. Increments of inflammatory mediators from the synovial joint or chondrocytes induce the expression of proteolytic enzymes such as MMPs, which leads to cartilage destruction (Sokolove & Lepus, 2013). To confirm whether PBSL could regulate the increase in inflammatory mediators under OA conditions, we examined the expression of inflammatory mediators in IL‐1β‐stimulated SW1353 cells and MIA‐induced mouse knee tissues. In SW1353 cells, the expression of inflammatory mediators [IL‐6, IL‐10, iNOS, cyclooxygenase 2 (COX2), and tumor necrosis factor‐α (TNF‐α)] increased after stimulation with IL‐1β. In contrast, pretreatment with PBSL suppressed the gene expression of IL‐6, IL‐10, iNOS, and TNF‐α, but the expression of COX2 was not altered (Figure 5a). Intriguingly, pretreatment with PBSL suppressed the expression of inflammatory mediators (IL‐6, IL‐10, iNOS, COX2, and TNF‐α) in the knee joint tissues of MIA‐stimulated mice (Figure 5b). Consistent with the gene expression results of experiments, pretreatment with PBSL suppressed the protein levels of IL‐6, COX2, and TNF‐α in SW1353 cells and the levels of COX2 and iNOS in mouse knee joint tissues (Figure 5c,d). These results suggest that PBSL may suppress the expression of inflammatory mediators in the pathological progression of OA.

### 3.6 Effects of pretreatment with PBSL‐derived components on NO synthesis in RAW264.7 cells

We have previously reported that PBSL comprises six ingredients, namely, benzoic acid, adenosine, uridine, hypoxanthine, adenine, and inosine (Lee et al., 2021). Therefore, we evaluated the level of NO synthesis in RAW264.7 cells to confirm the anti‐inflammatory effects of the components identified from PBSL. As shown in Figure 5e, NO generation was significantly increased by treatment with LPS (100 ng/mL), but NO synthesis was decreased in the benzoic acid, uridine, hypoxanthine, and inosine treatment groups. In particular, the inhibition rate of NO production was highest with 100 μM benzoic acid and uridine treatment, but significant results were only found for the benzoic acid treatment.

## 4 DISCUSSION

The global increase in OA prevalence due to the growth of the elderly population, increasing rate of obesity, and physical inactivity (Safiri et al., 2020) has urged extensive studies on the preventative or therapeutic approaches to OA (Roos & Arden, 2016; Sinusas, 2012; Walker‐Bone et al., 2000). Recent discoveries of preventive or therapeutic approaches to OA, coupled with therapeutic exercise or pharmacological therapies, have helped to reduce pain and inflammation in synovial joint tissue. However, current therapeutic approaches are inefficient in preventing or rescuing the progression of OA (Mobasheri, 2013; Zhang et al., 2016). Thus, novel pharmacological preventive and/or therapeutic drugs or functional foods derived from natural products are needed. Based on the ethnopharmacological uses of PBSL, the observations described here suggest that PBSL may be useful against OA. In this study, we demonstrated that pretreatment with PBSL alleviated physical disability and blocked subchondral bone remodeling in the MIA‐induced OA mouse model. Pretreatment with PBSL altered the expression of genes and proteins, which are involved in cartilage degradation and inflammation in chondrocytes and OA mouse models. According to the results of previous chemical profiling analysis, PBSL was identified as the six major components including adenine, hypoxanthine, uridine, adenosine, inosine, and benzoic acid (Lee et al., 2021). Among them, benzoic acid, the main component of PBSL, significantly suppressed the generation of NO. Identifying the mechanistic pathway by which benzoic acid suppresses NO synthesis to alleviate inflammation will provide further insight into the progression of OA and/or finding a novel therapeutic approach. Our findings revealed the novel efficacy of PBSL through chondroprotective effects and pathological progression in OA.

Pretreatment with PBSL rescued physical disability and subchondral bone remodeling in the MIA‐induced OA mouse model. Interestingly, this pretreatment also led to the maintenance of the cartilage layer and reduced microfractures in the subchondral bone. These results suggest that PBSL may be involved in the homeostasis of chondrocyte response to external and/or internal stress. Moreover, pretreatment with PBSL altered the expression of genes related to the progression of OA in mouse knee tissues.

MMPs and their inhibitors play an essential role in the downstream signaling pathways involved in articular cartilage damage and the pathological progression of OA. In the pathological progression of OA, the balance between MMPs and their inhibitors is important for pain progression and subchondral bone remodeling (Hayami et al., 2004; Shibakawa et al., 2005; Teng et al., 2019). Our in vivo results showed that pretreatment with PBSL reduced the expression of MMP3 and ADAMTs4. The degradation and remodeling of the cartilage matrix in arthritis are regulated by MMPs. Among the various MMPs, MMP3 and MMP13 mainly regulate the degradation of cartilage (Dean et al., 1989). MMP3 is secreted from chondrocytes and synovial cells in early OA and is closely associated with inflammation and articular cartilage damage in OA (Chen et al., 2014). These results suggested that pretreatment with PBSL suppressed the induction of MMP3 and ADAMTs4 response to MIA. Studies have illustrated that MMP13 is a novel therapeutic target for OA (Li et al., 2017; Wang et al., 2013). Although the expression of MMP13 was significantly decreased in the MIA‐induced OA and PBSL‐treated groups compared with the control group knee tissues, the expression of MMP13 was suppressed depending upon the concentration of PBSL in SW1353 cells. Interestingly, we observed that pretreatment with PBSL significantly induced TIMP1 expression in the mouse knee tissues; however, pretreatment with PBSL did not significantly induce TIMP1 expression in all in vitro groups. TIMPs are very well‐known endogenous inhibitors of MMPs and some members of the ADAMT families; they improve articular cartilage damage through the inhibition of MMPs and ADAMTs (Troeberg & Nagase, 2012). Thus, our data suggest that pretreatment with PBSL blocks the progress of OA through the suppressed expression of MMP3 and induced expression of TIMP1. Further studies are warranted to better understand the precise mechanism underlying PBSL effect on the regulation of MMP3 and TIMP1.

Our in vivo and in vitro data were not completely concordant; these differences might be attributed to differences in sample type between SW1353 cells and the mouse knees or differences in the type of stimuli used. SW1353 cells derived from female human chondrosarcoma have sufficient proliferative capacity and a similar response to modulating stimuli such as primary chondrocytes. However, Gebauer et al. (2005) demonstrated that the SW1353 cell line has limited potential to mimic the primary human chondrocyte response to IL‐1β stimuli. It has been demonstrated that key regulators in the progression of OA, such as MMPs, MMP inhibitors, and mediators of inflammation, are altered following stimulation (Pajak et al., 2017). For example, the expression of MMP3 and MMP9 is increased in early OA and decreases gradually after reaching its peak in the knees of MIA‐induced OA rats (Pajak et al., 2017). In contrast, the expression of TIMP1 oscillated after stimulation with MIA. In this study, mouse knee samples were harvested 10 days after MIA injection, whereas SW1353 cells were analyzed 24 h after IL‐1β stimulation, which could have affected the level of gene expression.

The occurrence or blocking of inflammation in the synovial joint is vital in the pathological progression of OA. Inflammation in the synovial tissues is closely related to cartilage degradation, and inflammation accelerates the induction of mediators, including iNOS and cytokines. These factors induce the production of NO, COX2, and MMPs, which leads to cartilage destruction (Huang et al., 2021). In LPS‐induced RAW264.7 cells, inflammatory factors such as NO and iNOS were reduced by treatment with PBSL. Our findings suggest that inhibiting the production of NO and iNOS may have a profound effect on the regulation of MMPs in OA chondrocytes. In addition, our results showed the anti‐inflammatory efficacy of PBSL and PBSL‐derived components by inhibiting the levels of NO and iNOS activation in LPS‐induced RAW264.7 cells. This suggests that PBSL is a valuable medicinal resource for OA therapy. Data on the NO inhibitory activity of benzoic acid and uridine are lacking, but the anti‐inflammatory effects via regulation of inflammatory factors in vitro and in vivo have been previously demonstrated (Cicko et al., 2015; Georgousaki et al., 2020; Jeengar et al., 2017; Schink et al., 2018; Trifilieff et al., 2002). Therefore, our results confirmed the possibility that the anti‐inflammatory effects of PBSL could be attributed to benzoic acid and uridine. Although benzoic acid did not significantly reduce the generation of NO, it significantly suppressed the generation of NO. This result suggests the possibility that other minor components of PBSL could also play a role in the regulation of NO synthesis. Hence, further identification and characterization of the other ingredients in PBSL are required.

Edible insects, which have been used for a long time in traditional medicine, are gaining more attention for their health benefit, this study demonstrated the functional value of PBSL for OA in terms of the usefulness of PBSL as an edible insect‐based herbal medicine. In addition, considering the characteristics of OA with complex and multiple factors, various attempts are being made to develop new treatment strategies for OA using modern high‐performance ‘omics’ scientific tools (including genomics, transcriptomics, proteomics, metabolomics, and microbiomics). Based on the results of a significant correlation between the gut microbiome and pain in chronic widespread diseases such as OA (Gonzalez‐Alvarez et al., 2023; Sánchez Romero, Meléndez Oliva, et al., 2021), further studies will be needed to elucidate the mechanism of action by the application of omics technologies.

In conclusion, our current results showed that PBSL treatment alleviated OA symptoms and has chondroprotective effects on OA progression in vivo and in vitro. These findings suggest that PBSL has value as edible insect that can be used in the development of functional foods for OA and/or associated symptoms. Therefore, PBSL may offer potential for application in clinical practice for protection against OA symptoms.

## AUTHOR CONTRIBUTIONS

**Jin Mi Chun:** Conceptualization (equal); data curation (equal); investigation (equal); methodology (equal); writing – original draft (equal); writing – review and editing (lead). **Hyeon‐Hwa Nam:** Data curation (equal); investigation (equal); methodology (equal); writing – original draft (equal). **Ji Hye Lee:** Conceptualization (equal); investigation (equal); methodology (equal); resources (equal). **Young Hye Seo:** Investigation (equal); resources (equal). **Hyo Seon Kim:** Resources (equal). **Byeong Cheol Moon:** Funding acquisition (lead); project administration (lead). **Jun Hong Park:** Conceptualization (equal); methodology (equal); writing – original draft (equal); writing – review and editing (equal).

## FUNDING INFORMATION

This research was funded by the Development of Sustainable Application for Standard Herbal Resources from the Korea Institute of Oriental Medicine (grant number KSN2013320 and KSN1822320).

## CONFLICT OF INTEREST STATEMENT

The authors declare no conflicts of interest.

## ETHICS STATEMENT

All animal experiments followed a protocol approved by the Institutional Animal Care and Use Committee of the Korea Institute of Oriental Medicine (KIOM 20‐028).

## Supporting information


Table S1.

## Data Availability

The data that support the findings of this study are available on request from the corresponding author.
