# Supplementary material for: Chondroprotective effects of Protaetia brevitarsis seulensis larvae as an edible insect on osteoarthritis in mice
Source: Food Sci Nutr. 2023 Oct 6;11(12):7887–99. doi: 10.1002/fsn3.3706 (PMC10724628; doi:10.1002/fsn3.3706)
Supplement: Supplementary file 1 — Table S1. [file FSN3-11-7887-s001.docx]

**Table S1. Primer sequences of real-time PCR analysis.**

| **Species** | **Primer Name** | **Sequence** | **Genebank** |
| --- | --- | --- | --- |
| Mouse | Actin-s | GGCTGTATTCCCCTCCATCG | NM_007393 |
|  | Actin-as | CCAGTTGGTAACAATGCCATGT |  |
|  | Mmp3-s | ACATGGAGACTTTGTCCCTTTTG | NM_010809 |
|  | Mmp3-as | TTGGCTGAGTGGTAGAGTCCC |  |
|  | Mmp9-s | CTGGACAGCCAGACACTAAAG | NM_013599 |
|  | Mmp9-as | CTCGCGGCAAGTCTTCAGAG |  |
|  | Timp1-s | GCAACTCGGACCTGGTCATAA | NM_001044384 |
|  | Timp1-as | CGGCCCGTGATGAGAAACT |  |
|  | Timp2-s | TCAGAGCCAAAGCAGTGAGC | NM_011594 |
|  | Timp2-as | GCCGTGTAGATAAACTCGATGTC |  |
|  | Adamts4-s | ATGGCCTCAATCCATCCCAG | NM_172845 |
|  | Adamts4-as | AAGCAGGGTTGGAATCTTTGC |  |
|  | Adamts5-s | GGAGCGAGGCCATTTACAAC | NM_011782 |
|  | Adamts5-as | CGTAGACAAGGTAGCCCACTTT |  |
|  | Il6-s | TAGTCCTTCCTACCCCAATTTCC | NM_031168 |
|  | Il6-as | TTGGTCCTTAGCCACTCCTTC |  |
|  | Il10-s | GCTCTTACTGACTGGCATGAG | NM_010548 |
|  | Il10-as | CGCAGCTCTAGGAGCATGTG |  |
|  | inos-s | GTTCTCAGCCCAACAATACAAGA | NM_010927 |
|  | inos-as | GTGGACGGGTCGATGTCAC |  |
|  | Cox2-s | TGAGCAACTATTCCAAACCAGC | NM_011198 |
|  | Cox2-as | GCACGTAGTCTTCGATCACTATC |  |
|  | Tnfα-s | CCCTCACACTCAGATCATCTTCT | NM_013693 |
|  | Tnfα-as | GCTACGACGTGGGCTACAG |  |
| Human | ACTIN-s | CATGTACGTTGCTATCCAGGC | NM_001101 |
|  | ACTIN-as | CTCCTTAATGTCACGCACGAT |  |
|  | MMP3-s | CTGGACTCCGACACTCTGGA | NM_002422 |
|  | MMP3-as | CAGGAAAGGTTCTGAAGTGACC |  |
|  | MMP9-s | TGTACCGCTATGGTTACACTCG | NM_004994 |
|  | MMP9-as | GGCAGGGACAGTTGCTTCT |  |
|  | MMP13-s | ACTGAGAGGCTCCGAGAAATG | NM_002427 |
|  | MMP13-as | GAACCCCGCATCTTGGCTT |  |
|  | TIMP1-s | CTTCTGCAATTCCGACCTCGT | NM_003254 |
|  | TIMP1-as | ACGCTGGTATAAGGTGGTCTG |  |
|  | TIMP2-s | GCTGCGAGTGCAAGATCAC | NM_003255 |
|  | TIMP2-as | TGGTGCCCGTTGATGTTCTTC |  |
|  | ADAMTs4-s | CTGGCACCTACCTGACTGG | NM_005099 |
|  | ADAMTs4-as | GTAACACGCCTAACAGGGCT |  |
|  | ADAMTs5-s | GAACATCGACCAACTCTACTCCG | NM_007038 |
|  | ADAMTs5-as | CAATGCCCACCGAACCATCT |  |
|  | IL6-s | CCTGAACCTTCCAAAGATGGC | NM_000600 |
|  | IL6-as | TTCACCAGGCAAGTCTCCTCA |  |
|  | IL10-s | TCAAGGCGCATGTGAACTCC | NM_000572 |
|  | IL10-as | GATGTCAAACTCACTCATGGCT |  |
|  | iNOS-s | TTCAGTATCACAACCTCAGCAAG | NM_000625 |
|  | iNOS-as | TGGACCTGCAAGTTAAAATCCC |  |
|  | COX2-s | CTGGCGCTCAGCCATACAG | NM_000963 |
|  | COX2-as | CGCACTTATACTGGTCAAATCCC |  |
|  | TNFα-s | GAGGCCAAGCCCTGGTATG | NM_000594 |
|  | TNFα-as | CGGGCCGATTGATCTCAGC |  |

**Table S2. Primary antibodies for western blotting**

| **Antibodies** | **Corporation** | **Catalog No.** |
| --- | --- | --- |
| MMP3 | Cell signaling | 14351 |
| MMP9 | Cell signaling | 13667 |
| MMP13 | Abcam | Ab39012 |
| ACTIN | Sigma | A3853 |
| TIMP1 | Cell signaling | 8946 |
| TIMP2 | Cell signaling | 5738 |
| TIMP3 | Cell signaling | 5673 |
| iNOS | Cell signaling | 13120 |
| IL6 | Santa Cruz Biotechnology | sc-1265 |
| COX2 | Santa Cruz Biotechnology | sc-1745 |
| TNF-α | Santa Cruz Biotechnology | sc-1350 |
